# High levels of circulating CD34+ cells at autologous stem cell collection are associated with favourable prognosis in multiple myeloma

**DOI:** 10.1038/bjc.2011.329

**Published:** 2011-08-30

**Authors:** J Raschle, D Ratschiller, S Mans, B U Mueller, T Pabst

**Affiliations:** 1 Department of Medical Oncology, University Hospital and University of Berne, Berne, Switzerland; 2 Department of Internal Medicine, University Hospital and University of Berne, Berne, Switzerland

**Keywords:** myeloma, autologous, transplantation, stem cells, CD34, prognosis

## Abstract

**Background:**

High-dose chemotherapy with autologous stem cell transplantation is a cornerstone in the first-line treatment of multiple myeloma patients. However, only few factors have been identified affecting the outcome in such patients. We hypothesised that varying levels of mobilised CD34+ cells confer prognostic information in myeloma patients undergoing high-dose chemotherapy.

**Methods:**

We determined circulating CD34+ cells at the day of peripheral stem cell collection in 158 consecutive myeloma patients between January 2001 and August 2010. Patients were stratified into two groups (super *vs* normal mobilisers) with a cutoff of 100 000 peripheral CD34+ cells per ml.

**Results:**

We found that patients with more than 100 000 peripheral CD34+ cells per ml had a better overall survival (*P*=0.005) and a prolonged time to progression (*P*=0.0398) than patients with CD34+ cell counts below 100 000 CD34+ cells per ml. High levels of CD34+ cells were an independent marker for better overall survival and time to progression in a multivariate analysis that included disease stage, response at transplant, light-chain subtype, age, sex, and height.

**Conclusion:**

Our results suggest that high levels of mobilised peripheral CD34+ cells are associated with favourable outcome in myeloma patients undergoing autologous transplantation.

A variety of factors have been reported to affect prognosis in patients with multiple myeloma including cytogenetic abnormalities, molecular markers, cytokine profiling, and clinical parameters (Kyle and Rajkumar, 2009; Rajkumar, 2009; Barlogie *et al*, 2010; Dingli and Rajkumar, 2010; Munshi *et al*, 2011). In particular, clinical features such as high lactate dehydrogenase levels, IgA subtype, presence of extramedullary disease, renal failure, high levels of serum-free light chains or of the serum *κ*/*λ* free light-chain ratio, plasmablastic disease, or presentation as primary plasma cell leukaemia have been identified to confer unfavourable prognostic information (Munshi *et al*, 2011). Accordingly, risk stratification models have been established in multiple myeloma (Greipp *et al*, 2005; Rajkumar and Kyle, 2005; Kyle and Rajkumar, 2009; Rajkumar, 2010).

With growing insights into the genetic heterogeneity of multiple myeloma, additional prognostic factors have been proposed allowing stratification of myeloma patients into different risk categories, possibly paving the way towards a more risk-adapted therapeutic approach (Rajkumar, 2009). Such an evolving risk assessment can be based on molecular and cytogenetic abnormalities detected by conventional karyotyping, fluorescent *in situ* hybridisation, and/or gene expression profiling (Fermand *et al*, 2005; Greipp *et al*, 2005; Rajkumar and Kyle, 2005; Kyle and Rajkumar, 2009; Chang *et al*, 2010; Hose *et al*, 2011; Munshi *et al*, 2011).

High-dose chemotherapy followed by autologous stem cell transplantation is a cornerstone within the current standard treatment for symptomatic myeloma patients fit for intensive treatment (Child *et al*, 2003; Terpos *et al*, 2003; Barlogie *et al*, 2004; Blade *et al*, 2005; Fermand *et al*, 2005; Rajkumar and Kyle, 2005; Wang *et al*, 2007; Palumbo *et al*, 2009; Rajkumar, 2009; Lonial, 2010; Cavo *et al*, 2011). In fact, a number of studies have established the benefit of autologous transplantation for myeloma patients in prolonging the time to progression and, at least in some of them, also in improving overall survival (Attal *et al*, 1996; Child *et al*, 2003; Barlogie *et al*, 2004; Blade *et al*, 2005; Cavo *et al*, 2011).

In this retrospective study, we investigated the level of circulating CD34+ cells at the day of peripheral stem cell collection as a prognostic marker in myeloma patients. We hypothesised that excellent stem cell mobilisation is associated with an intact bone marrow homeostasis and thus confers favourable prognostic information. In fact, we found that levels of circulating CD34+ cells below 100 000 per ml at the day of stem cell collection were associated with shorter time to progression and overall survival.

## Study design

### Patients

A total of 158 consecutive myeloma patients underwent stem cell collection with subsequent autologous stem cell transplantation as a component of their first-line treatment between January 2001 and August 2010 at the Department of Medical Oncology, University Hospital in Bern, Switzerland. Clinical characteristics at diagnosis and mobilisation, regimens used for induction and mobilisation, and the course of the disease of the study population are summarised in Table 1 and Supplementary Figure S1. All patients had G-CSF in addition to chemotherapy for mobilisation. Chemotherapy was high-dose cyclophosphamide until December 2005 and vinorelbine since January 2006 (Bargetzi *et al*, 2003). No patient received a CXCR4 antagonist, and no CD34 selection was performed.

### Statistical analysis

Patients were stratified into one group with more than 100 000 peripheral CD34+ cells per ml (super mobilisers), and a group with less than 100 000 circulating CD34+ cells per ml (normal mobilisers) at the day of apheresis. Overall survival was defined as the time from the day of stem cell harvest until death or last follow-up whichever occurred first. The time until first progression was the time from the day of apheresis until first progression or death, whichever occurred earlier, or until last follow-up if the patient remained in remission. Curves depicting overall survival and time to progression were performed using the Kaplan–Meier method. The survival analysis was performed using log-rank test. To evaluate the effects of parameters on outcome, the two groups were compared using the *χ*^2^-test or Fisher's exact test, and differences in the mean values in case of continuous variables were tested using *t*-test. The Cox proportional hazard regression was applied to analyse various risk factors on survival. Results were considered significant if the *P*-value was below 0.05. All statistical analyses and graphs were performed using graph pad prism program 5.04 (1992–2010; GraphPad Software, Inc., La Jolla, CA, USA) and Statview 5.0.1 (SAS Institute, Cary, NC, USA).

## Results

A total of 158 consecutive myeloma patients undergoing autologous transplantation during their first-line treatment were stratified into two groups based on the level of circulating peripheral CD34+ cells at the day of stem cell collection. In all, 69 patients (super mobilisers) had more than 100 000 CD34+ cells per ml, whereas 89 patients had less than 100 000 CD34+ cells per ml (normal mobilisers). The individual values of all patients are depicted in a Supplementary Figure S2. The two groups showed no differences with regards to sex, age, light-chain subtype, stage at diagnosis, cytogenetics, height, weight, radiotherapy before stem cell collection, response to induction treatment, single *vs* tandem transplantation, time between apheresis and transplantation, or regimens used for induction or mobilisation (Table 1). The mean number of circulating CD34+ cells at the day of stem cell collection in the super mobiliser group was 179 609 CD34+ cells per ml, and 44 381 CD34+ cells per ml in the normal mobiliser group. The total number of CD34+ cells collected at apheresis was higher in the super mobiliser group (18.17 × 10^6^ per kg *vs* 10.37 × 10^6^ per kg; *P*=<0.0001), and patients in the super mobiliser group received more CD34+ cells at autologous transplantation (5.27 × 10^6^ per kg *vs* 3.56 × 10^6^ per kg; *P*=0.0055).

**Figure 1:**
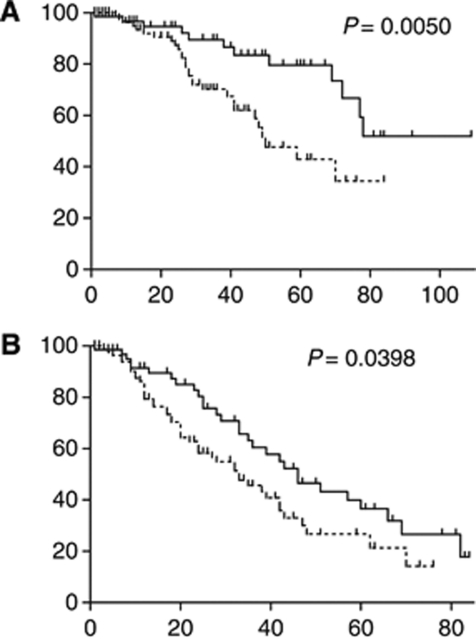
Better overall survival and longer time to progression in super mobiliser (*n*=69) *vs* normal mobiliser myeloma patients (*n*=89). Kaplan–Meier curves are depicted for overall survival (**A**) and time to progression (**B**) comparing the super mobiliser group (continued line) with the group of normal mobilisers (dotted line). A value of 100 000 CD34+ cells per ml circulating at the day of stem cell collection was used to stratify between the two groups. *x* axis in months, *y* axis depicts percent survival. (**A**) Median OS in the super mobiliser group was not reached; median OS in the normal mobiliser group was 50 months. (**B**) A total of 70 patients had a progression, including 27 patients in the super mobiliser group with a median TTP of 46 months compared with 43 patients in the normal mobiliser group with a median TTP of 33 months.

After a mean follow-up of 32.46 months, 42 patients have died, with 12 deaths occurring in the super mobiliser and 30 in the normal mobiliser group (*P*=0.0289). The median overall survival of all myeloma patients was 72 months (Supplementary Figure S1). Although the group of super mobilisers did not yet reach the median survival, the group of normal mobilisers had a median survival of 50 months (*P*=0.0050; Figure 1).

A total of 70 patients in our cohort had a first progression of their disease after autologous transplantation, with 27 patients progressing in the super mobiliser and 43 patients in the normal mobiliser group. The group of super mobilisers showed a longer time to progression, with a median time to progression of 46 months compared with 33 months in the normal mobiliser group (*P*=0.0398).

The favourable effect of high levels of circulating CD34+ cells at the day of stem cell collection was observed independent from the type of chemotherapy regimen used for induction, and it was also independent from the chemotherapy (cyclophosphamide or vinorelbine) used for mobilisation (data not shown). Better OS and TTP in the group of super mobilisers were also observed across the ISS stages, with the favourable effect reaching significance (*P*=0.0039 and *P*=0.011) for patients with ISS stage III at diagnosis (data not shown). We did not observe that the number of CD34+ cells infused at transplantation-affected OS or TTP, with *P*=0.754 and *P*=0.899, respectively, for patients below *vs* above the mean value of infused CD34+ cells (data not shown).

In a multivariate analysis, the level of circulating CD34+ cells turned out to be an independent prognostic factor for OS (*P*=0.0011) and TTP (*P*=0.0228). This multivariate analysis also included light-chain subtype, sex, age, height, and disease stage at diagnosis, as well as the type of response at transplant (Table 2).

## Discussion

To our knowledge, this is the first report identifying varying levels of circulating CD34+ cells at the day of stem cell collection to be a prognostic marker in myeloma patients. Although previous studies indicated that patients with various lymphoid malignancies mobilising large numbers of CD34+ cells (‘super mobilisers’) enjoy improved survival following autologous stem cell transplantation (Stockerl-Goldstein *et al*, 2000; Gordan *et al*, 2003; Bolwell *et al*, 2007; Hiwase *et al*, 2008), such data are lacking so far for myeloma patients. A small study including 39 myeloma patients found no difference in outcome (Kakihana *et al*, 2010). As the two groups of super *vs* normal mobilisers in our cohort did not differ in clinical characteristics, we can exclude one or several of such parameters, to have affected the conclusion of this analysis. With regards to the retrospective character of this study, we consider a prospective evaluation of the effect of levels of mobilised CD34+ cells on outcome to be desirable, and we are in the process of initiating such a study.

The reason for the better clinical course of myeloma patients with large numbers of circulating CD34+ cells at the day of stem cell collection remains to be elucidated. One hypothesis is that patients with a high number of circulating CD34+ cells might have ‘intact’ stem cell niches with conservation of the number of stem cells and their regulation of self-renewal and differentiation (Scadden, 2006). This intact stem cell niche status might enable such patients to mobilise large numbers of CD34+ cells during the stem cell stimulation procedure (Wilson and Trumpp, 2006). Bone marrow infiltration by malignant plasma cells at diagnosis or at stem cell collection might serve as a surrogate marker for altered stem cell homeostasis. However, we observed no difference in the mean bone marrow infiltration between the groups of super *vs* normal mobilisers (data not shown).

Another factor possibly affecting the conclusion of this study is the number of CD34+ cells used at autologous transplantation. Patients in the super mobiliser group in this study received higher numbers of CD34+ cells during autologous transplantation (*P*=0.0055). In fact, the composition of the infused cellular products, such as the number of lymphocytes and/or dendritic cells, has been reported to affect the outcome in allogenous transplantation in patients with lymphoid malignancies (Bolwell *et al*, 2007). However, we did not observe in our cohort of myeloma patients undergoing autologous transplantation that the number of CD34+ cells infused at transplantation affected OS or TTP, with *P*=0.754 and *P*=0.899, respectively, for patients below *vs* above the mean value of infused CD34+ cells. In a Supplementary Table S1, we also included the number of infused CD34+ cells in the multivariate analysis. We found that the number of circulating CD34+ cells still remained an independent factor for OS with a *P-*value of 0.0004. We thus conclude that the favourable effect of high numbers of circulating CD34+ cells is independent from the number of infused CD34+ cells at autologous transplantation in myeloma patients.

In conclusion, this study identified high levels of circulating CD34+ cells at the day of stem cell collection to be associated with favourable outcome in myeloma patients undergoing autologous transplantation. We propose that this biomarker might be considered to be integrated into future risk stratification in myeloma patients to select patients for a post-transplant maintenance or consolidation strategy.

## Figures and Tables

**Table 1:** Patient characteristics, mobilisation treatment and autologous transplantation

	**Super mobilisers**	**Normal mobilisers**	**All**	***P*-value**
*n*	69	89	158	
				
*Age at diagnosis (years)*				
Mean±s.e.m.	55.42±0.8917	56.52±0.7661	56.04±0.5810	
Range	32–71	30–69	30–71	
				
*Cytogenetics* a				
Not done	53	65	118	
Done	16	24	40	
Normal	5	8	13	
del13q	11	13	24	
t(11;14)	1	0	1	
del17p	1	2	3	
+3/+7/+9	0/0/0	1/2/1	1/2/1	
t(4;14)	0	3	3	
				
*Sex*				
Male/female	46/23	63/26	109/49	
				
*Light chain* b				
*κ*/*λ*	44/23	58/28	102/51	
				
*Stage at diagnosis (ISS)* c				
I/II/III	15/17/32	22/26/38	37/43/70	
				
*Subtypes* d				
IgG/IgA	47/10	59/14	106/24	
Light chain only	6	10	16	
Asecretory	3	2	5	
Mean follow-up (months)	35.84	29.83	32.46	
				
*Progression*				
Yes/no	27/42	43/46	70/88	
				
*Dead* e				
Yes/no	12/57	30/59	42/116	0.0289
				
*Median time between apheresis and transplantation (d)*				
Mean±s.e.m.	32.46±4.938	26.20±2.247	28.94±2.503	
Range	7–242	8–100	7–242	
				
*First-line treatment* f				
1 line/>1line	52/17	72/17	124/34	
				
*First-line treatment*				
VAD	36	45	81	
Bortezomib/dex.	17	27	44	
Thalidomide/dex.	11	8	19	
dex.	3	6	9	
Melphalan/pred.	2	3	5	
Single/tandem transplantation	29/40	25/64	54/104	
				
*Response to induction* g				
Complete remission	4	7	11	
VGPR	12	16	28	
Partial remission	51	62	113	
Stable disease	1	3	4	
				
*Radiotherapy before stem cell collection*				
Yes/no	10/59	16/73	26/132	
				
*Mobilisation chemotherapy*				
Vinorelbine	34	48	82	
Cyclophosphamide	33	30	63	
Bortezomib/dex.	0	9	9	
VAD	2	2	4	
				
*Peripheral leukocytes at day of stem cell collection (g l* ^−*1*^ *)*				
Mean±s.e.m.	25.76±1.874	16.56±1.165	20.58±1.107	<0.0001
Range	4.1–52.7	1.2–52.7	1.2–52.7	
				
*Circulating peripheral CD34+ cells at day of stem cell collection (cells per ml)*				
Mean±s.e.m.	179 609±10 937	44 381±2602	103 436±7309	<0.0001
Range	103 740–608 760	2800–99 120	2800–608 760	
				
*Total number of collected CD34+ cells (cells kg* ^*−1*^ *)*				
Mean±s.e.m.	18.17±1.022	10.37±0.5541	13.78±0.6243	<0.0001
Range	2.4–49.4	2.04–26.35	2.04–49.4	
				
*CD34+ cells re-infused (cells kg* ^*−1*^ *)*				
Mean±s.e.m.	5.773±0.2419	4.381±0.4	4.824±0.1625	0.0055
Range	2.3–12.6	2.01–10	2.01–12.6	
				
*Neutrophil engraftment (days)*				
Mean±s.d.	11±0.2698	11.26±0.2615	11.14±0.1882	
Range	1–17	4–18	1–18	

Abbreviations: del=deletion; dex.=dexamethasone; Ig=immunoglobulin; ISS=international staging system; pred.=prednisone; t=translocation; VAD=vincristine, adriamycin, dexamethasone; VGPR=very good partial response.

a Some of the patients had several cytogenetic abnormalities.

b The information on the light-chain subtype was not available in five patients.

c The information on the ISS stage at diagnosis in eight patients.

d The information on subtype in seven patients.

e Causes of death were all due to myeloma progression, with the exception of three patients in the normal mobiliser group (heart failure; infection; suicide; one patient each) and one patient in the super mobiliser group (infection).

f No patient had a first-line treatment with lenalidomide.

g The information on the response to induction in two patients.

**Table 2:** Multivariate analysis for overall survival and progression-free survival

			**Confidence interval of hazard ratio**	
	***P*-value**	**Hazard ratio**	**Lower 95%**	**Upper 95%**
*Overall survival*				
Light chain (*κ* *vs* *λ*)	0.0159	1.912	1.009	3.002
Sex (male *vs* female)	0.8444	1.112	0.622	1.925
CD34 + cells (super *vs* normal mobilisers)	0.0011	4.382	1.973	9.288
Age (> *vs* < mean age)	0.9886	0.980	0.552	1.801
Height (> *vs* < mean height)	0.7885	1.282	0.675	2.442
CR and VGPR *vs* PR and SD	0.0164	3.656	1.608	6.084
ISS stage (III *vs* I and II)	0.2922	0.695	0.358	1.312
				
*Time to progression*				
Light chain (*κ* *vs* *λ*)	0.0422	1.751	1.006	2.944
Sex (male *vs* female)	0.5205	1.185	0.724	2.002
CD34 + cells (super *vs* normal mobilisers)	0.0228	1.884	1.061	3.012
Age (> *vs* < mean age)	0.7488	0.892	0.622	1.382
Height (> *vs* < mean height)	0.7880	0.912	0.572	1.533
CR and VGPR *vs* PR and SD	0.0330	3.926	1.722	5.258
ISS stage (III *vs* I and II)	0.1566	0.652	0.423	1.284

Abbreviations: CR=complete remission; PR=partial remission; SD=stable disease; VGPR=very good partial remission. Multivariate analysis investigating overall survival and time to progression using the Cox proportional-hazard regression model.
