# Supplementary material for: High levels of circulating CD34+ cells at autologous stem cell collection are associated with favourable prognosis in multiple myeloma
Source: Br J Cancer. 2011 Aug 30;105(7):970–4. doi: 10.1038/bjc.2011.329 (PMC3185945; doi:10.1038/bjc.2011.329)
Supplement: Supplementary Figures S1 and S2 [file bjc2011329x1.ppt]

## Slide 1
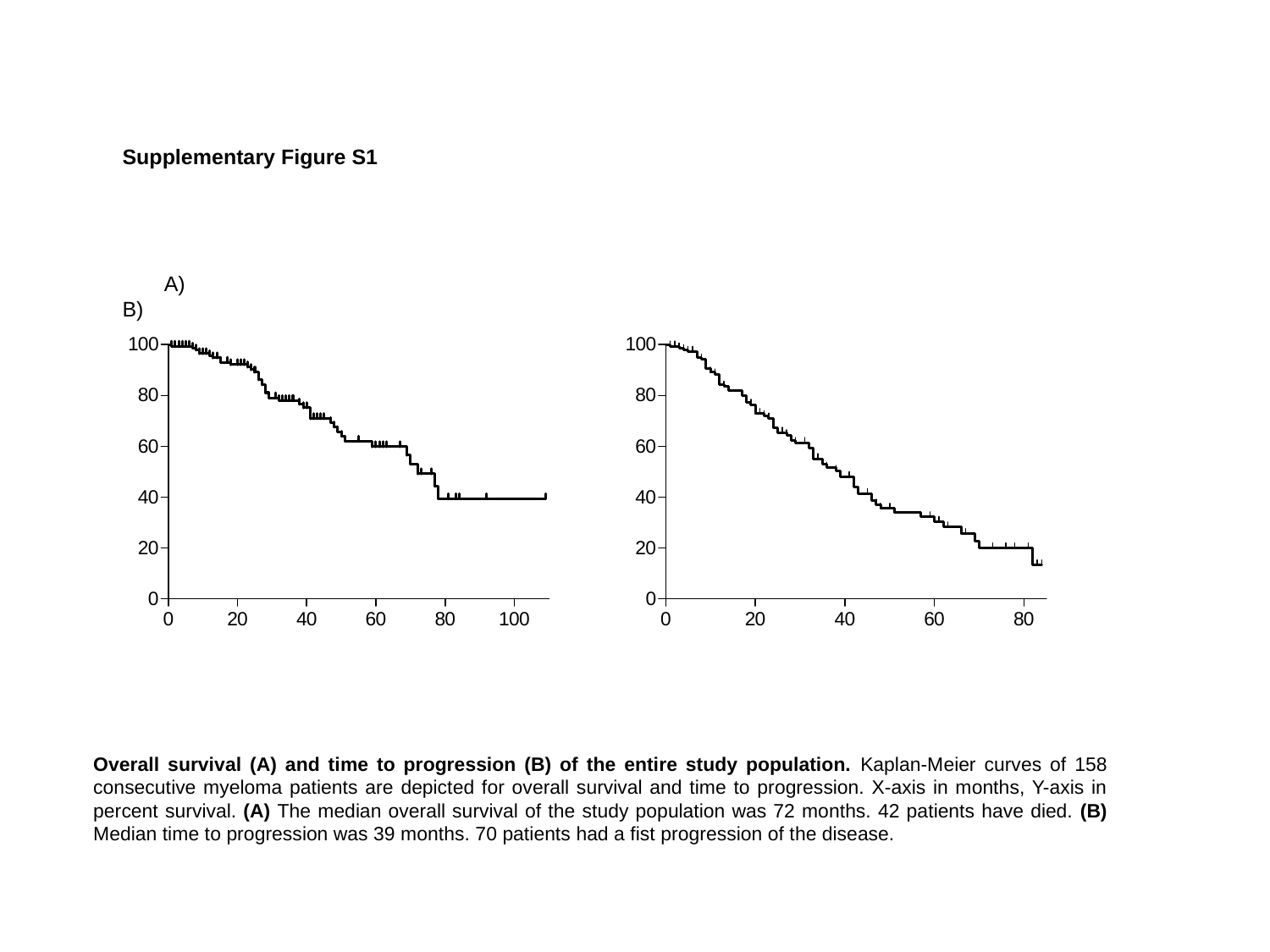

# Supplementary Figure S1 A)								 B)
Overall survival (A) and time to progression (B) of the entire study population. Kaplan-Meier curves of 158 consecutive myeloma patients are depicted for overall survival and time to progression. X-axis in months, Y-axis in percent survival. (A) The median overall survival of the study population was 72 months. 42 patients have died. (B) Median time to progression was 39 months. 70 patients had a fist progression of the disease.

## Slide 2
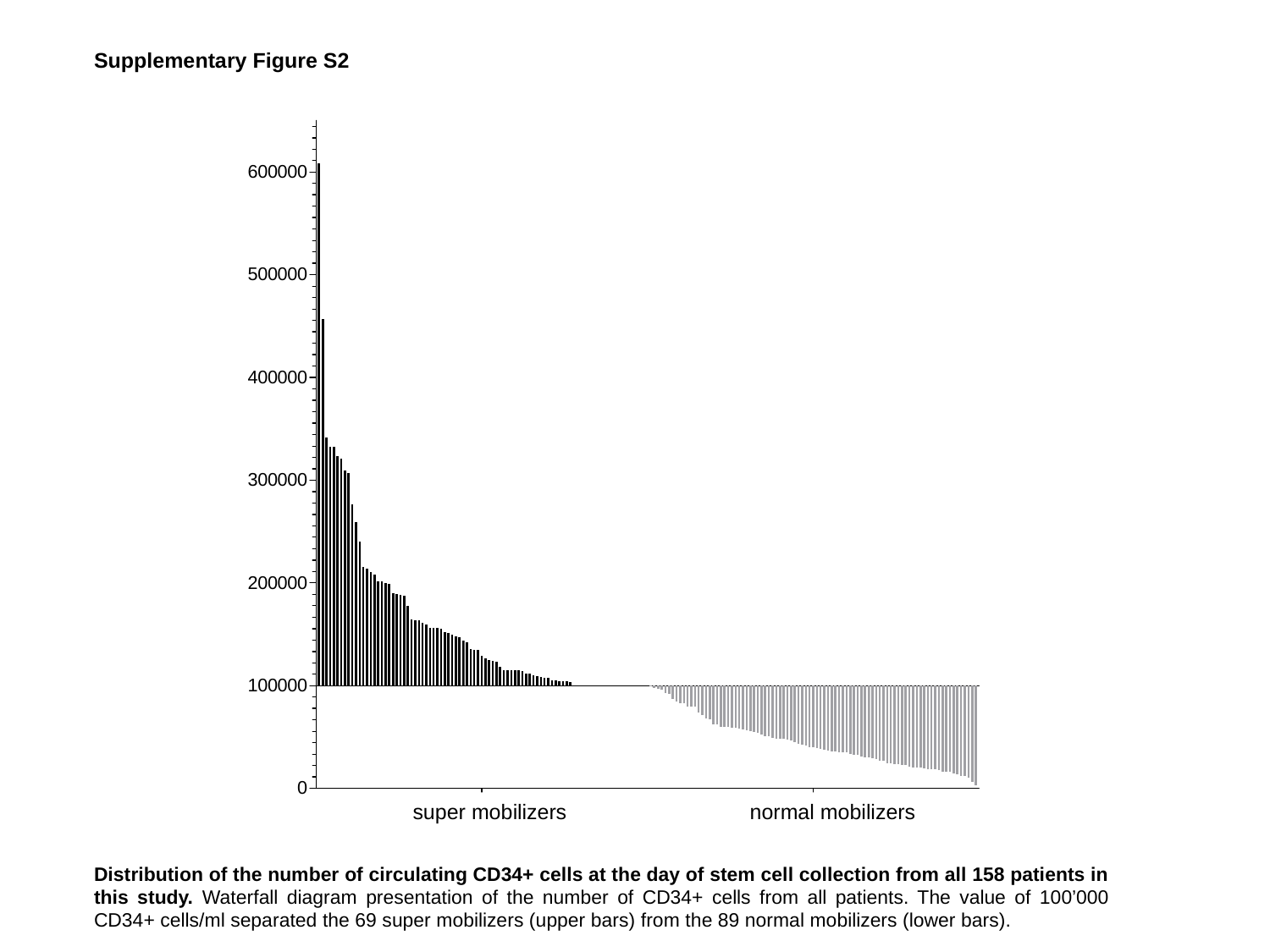

# Supplementary Figure S2
super mobilizersn
normal mobilizers
Distribution of the number of circulating CD34+ cells at the day of stem cell collection from all 158 patients in this study. Waterfall diagram presentation of the number of CD34+ cells from all patients. The value of 100’000 CD34+ cells/ml separated the 69 super mobilizers (upper bars) from the 89 normal mobilizers (lower bars).
