# Supplementary material for: High levels of circulating CD34+ cells at autologous stem cell collection are associated with favourable prognosis in multiple myeloma
Source: Br J Cancer. 2011 Aug 30;105(7):970–4. doi: 10.1038/bjc.2011.329 (PMC3185945; doi:10.1038/bjc.2011.329)
Supplement: Supplementary Table S1 [file bjc2011329x2.doc]

| Supplementary Table S1: |  |  |  | |
| --- | --- | --- | --- | --- |
|  |  |  |  | |
|  |  |  | Confidence Interval of Hazard Ratio | |
|  |  |  |  |  |
|  | *P*-value | Hazard Ratio | Lower 95% | Upper 95% |
|  |  |  |  |  |
| overall survival |  |  |  |  |
| light chain (kappa vs lambda) | .0200 | 2.244 | 1.136 | 4.432 |
| sex (male vs female) | .7337 | 1.168 | 0.477 | 2.865 |
| CD34+ cells (super vs normal mobilizers) | .0004 | 4.676 | 2.006 | 10.899 |
| age (> vs < mean age) | .9586 | 0.9820 | 0.501 | 1.924 |
| height (> vs < mean height) | .4706 | 1.362 | 0.589 | 3.150 |
| infused CD34 + cells (> vs < mean) | .2979 | 0.673 | 0.319 | 1.417 |
| ISS stage (III vs I and II) | .2736 | 0.686 | 0.349 | 1.347 |
|  |  |  |  |  |

Multivariate analysis investigating overall survival using the Cox proportional-hazard regression model.
